# Do measures matter? Comparing surface-density-derived and census-tract-derived measures of racial residential segregation

**DOI:** 10.1186/1476-072X-9-29

**Published:** 2010-06-12

**Authors:** Michael R Kramer, Hannah L Cooper, Carolyn D Drews-Botsch, Lance A Waller, Carol R Hogue

**Affiliations:** 1 Department of Epidemiology, Rollins School of Public Health, Emory University, Atlanta, GA, USA; 2 Department of Behavioral Sciences and Health Education, Rollins School of Public Health, Emory University, Atlanta, GA, USA; 3 Departments of Biostatistics, Rollins School of Public Health, Emory University, Atlanta, GA, USA

## Abstract

**Background:**

Racial residential segregation is hypothesized to affect population health by systematically patterning health-relevant exposures and opportunities according to individuals' race or income. Growing interest into the association between residential segregation and health disparities demands more rigorous appraisal of commonly used measures of segregation. Most current studies rely on census tracts as approximations of the local residential environment when calculating segregation indices of either neighborhoods or metropolitan areas. Because census tracts are arbitrary in size and shape, reliance on this geographic scale limits understanding of place-health associations. More flexible, explicitly spatial derivations of traditional segregation indices have been proposed but have not been compared with tract-derived measures in the context of health disparities studies common to social epidemiology, health demography, or medical geography. We compared segregation measured with tract-derived as well as GIS surface-density-derived indices. Measures were compared by region and population size, and segregation measures were linked to birth record to estimate the difference in association between segregation and very preterm birth. Separate analyses focus on metropolitan segregation and on neighborhood segregation.

**Results:**

Across 231 metropolitan areas, tract-derived and surface-density-derived segregation measures are highly correlated. However overall correlation obscures important differences by region and metropolitan size. In general the discrepancy between measure types is greatest for small metropolitan areas, declining with increasing population size. Discrepancies in measures are greatest in the South, and smallest in Western metropolitan areas. Choice of segregation index changed the magnitude of the measured association between segregation and very preterm birth. For example among black women, the risk ratio for very preterm birth in metropolitan areas changed from 2.12 to 1.68 for the effect of high versus low segregation when using surface-density-derived versus tract-derived segregation indices. Variation in effect size was smaller but still present in analyses of neighborhood racial composition and very preterm birth in Atlanta neighborhoods.

**Conclusion:**

Census tract-derived measures of segregation are highly correlated with recently introduced spatial segregation measures, but the residual differences among measures are not uniform for all areas. Use of surface-density-derived measures provides researchers with tools to further explore the spatial relationships between segregation and health disparities.

## Background

Throughout the twentieth century residential segregation by race and class has been described by sociologists [1-3]. W.E.B. DuBois [4] detailed differences in black mortality by residential neighborhood in Philadelphia, and in the 1950's Yankauer [5] explicitly restated residential segregation as a public health concern. More recently segregation has reemerged as a possible "fundamental determinant" [6] of racial disparities in such health outcomes as all-cause mortality [7], preterm birth [8,9], self-rated health [10], obesity [11], survival for individuals with end stage renal disease[12] and stage at cancer diagnosis [13].

Residential segregation can be conceptualized as a descriptive state or condition, or as an active process [14]. As an adjective, 'segregated' describes the degree to which there is departure from a random spatial distribution of racial, ethnic, or economic groups within a city or metropolitan area. Alternatively 'segregate' as a verb evokes an active process of differential sorting of individuals into residential environments, thereby influencing their probability of experiencing a range of place-related exposures. Both the condition and the process of segregation may represent geographically variable operationalizations of structural inequality or institutionalized racism, which may be toxic to health, particularly of minority groups [6].

In the health literature, the term 'racial segregation' has been evoked to describe processes and health determinants at two distinct geographic scales. Although these scales are related, the implied mechanisms by which segregation affects health may be quite different. One line of research, extending from the neighborhood effects literature, evaluates the role of the racial/ethnic composition or density in the neighborhood of residence. Some investigators have hypothesized that residents of predominantly minority neighborhoods disproportionately experience negative health effects resulting from neighborhood deprivation, infrastructure disinvestment, or decreased access to health promoting resources including healthy food choices and walkable neighborhoods [15-18]. Other investigators have posited that living in neighborhoods with large minority populations is health-protective for minority residents due to the buffering effects of social networks and support in racial/ethnic enclaves [19,20].

While the local neighborhood environment is a critical component of understanding health effects of residential segregation, many researchers argue that the social processes of interest do not operate solely within neighborhoods but across a broader geographic context [21]. For instance metropolitan areas encompass the urban and suburban areas across which the phenomenon of residential segregation occurs, while also capturing economically and socially integrated areas that may have spatially varying economic opportunity [22]. Health research at this broader geographic scale may posit that segregation is not an exposure limited to residents of a few neighborhoods, but rather a larger context in which all minority residents have some degree of exposure [23].

With increasing focus on the association between residential segregation and health, researchers have called for a more rigorous and nuanced approach to conceptualizing and measuring residential settlement patterns [21,24]. For research focused on racial composition primarily within neighborhoods, the most common measure is the minority population percent in a given areal unit (e.g. census tract, zip code area, county). This simple measure may be analyzed as a continuous variable assuming linear relationships [25], or acknowledging possible threshold effects of racial composition, it may be analyzed as a binary or categorical variable [26,27].

More measurement tools are available for researchers interested in geographic scales broader than neighborhoods (e.g. Metropolitan Statistical areas Areas, MSAs). Of five axes or dimensions of segregation patterns described by Massey and Denton, three are commonly employed in research on segregation and health: evenness (the degree to which social groups are similarly distributed across areal units in a metropolitan region); exposure/isolation (the likelihood for inter-group interaction within local areal units); and clustering (the proximity of areal units with high minority concentration to one another within a metropolitan region) [28].

Whether segregation is measured as an attribute of individual neighborhoods or as an aggregate pattern across all neighborhoods in a metropolitan area, measures of segregation depend on operationalizing the 'residential neighborhood' that corresponds to the spatial scale at which health-relevant social and economic exposures operate. The most common definition is the census tract, which is a well-defined areal unit that ideally has a homogeneous population of approximately 1500-8000 residents [29]. However this definition has been critiqued on the basis of the modifiable areal unit problem (MAUP) wherein quantitative results based on data aggregated to small areas depends on both the level of aggregation and which set of small areas are used. Arbitrary changes in tract boundaries could result in different values of measured segregation without real change in residential location [30,31]. The use of census tracts to proxy neighborhoods presumes that all residents of a tract have a uniform experience of diversity or economic opportunity, that boundaries between tracts approximate meaningful social or economic boundaries and that residential patterns at smaller or larger scales are not meaningfully different (or relevant) from those at the scale of the tract. These assumptions may be false generally, but the implications could also vary by region of the country or by relative size of metropolitan areas because of historical patterns of urban development in different regions. Patterns of both residential segregation and health disparities have been noted to vary by geographic region and metropolitan size [32].

In response to these criticisms, Reardon & O'Sullivan proposed a new class of spatial segregation indices which allow a flexible definition of local neighborhood applied to a continuous population density surface across metropolitan space [33]. Their approach proposes estimating residential segregation using an egocentric neighborhood definition, where the local environment of each residence is uniquely defined as the area surrounding that residence, and further allowing the size of that area to be researcher-specified and not constrained to the scale of census geography. To date the relative merit of these new measures of segregation have not been compared to traditional tract-derived measures. Furthermore, the potential effect of exposure misclassification bias resulting from choice of measure in epidemiologic models has not been considered in the context of segregation-health associations.

The goal of this study is to compare tract-derived and spatial surface-density-derived measures of segregation as they might be used in a) studies which focus on segregation within MSAs; and b) studies which focus on racial composition as a neighborhood-level effect. Of particular interest is the possibility for meaningful exposure misclassification or mis-measurement in epidemiologic studies interested in the effects of varying degrees of residential segregation on health outcomes. Previous research suggests there is an association between residential segregation and risk of preterm birth, particularly for black women [8,34,35]. Therefore, to motivate the previous questions, measures of segregation were linked to birth records to assess differences in the magnitude of the segregation-preterm birth association and goodness of model fit using alternate indices of segregation.

Residential segregation can occur across different economic, racial, and ethnic groups. Acknowledging that most segregation-health research in the United States is focused on black-white segregation, and that many of the largest health disparities in the US are between blacks and whites, we focus this analysis on this particular case.

## Methods

### Measuring MSA segregation

Following Massey and Denton, we measured evenness segregation with the dissimilarity index, and exposure/isolation with the black isolation index (xP*x), in each case using census tracts as approximations of the local residential neighborhood. The formulas follow using the notation of Reardon and O'Sullivan [33]:(1)

Tract-derived dissimilarity index(2)

Tract-derived black isolation index

For each equation, i indexes n census tracts within an MSA. *π *is the proportion black either in the census tract (*π*_*i*_) or in the MSA overall (*π*). T is the total (black plus white) population count of the MSA, and t_i _is the total population count for the i^th ^census tract. In each case the measure is a population-weighted average across all tracts. Each index ranges from 0 (least segregated) to 1 (most segregated). The dissimilarity index compares the racial composition of each tract to the overall MSA composition and approximates the proportion of blacks who would have to move to a different tract to produce even racial distribution across tracts. The black isolation index approximates the probability that any two randomly drawn individuals from the same tract are both black.

We calculated the surface-density-derived measures using an ArcGIS script developed for the purpose [36]. Following Lee, et al, [30] we converted census block data for white, black, and total population counts to a surface density on a fine grid (50 m × 50 m). Census blocks are the highest resolution population data available. In the Atlanta MSA the median area of census *tracts *is 1,372 acres, while the median area of census *blocks *is just 9.6 acres. To more realistically approximate the population distribution in space, we employed a pycnophylactic (mass preserving) smoothing process to reduce abrupt changes in density at block boundaries without artificially shifting population mass across boundaries [37]. All possible locations within a given MSA were thus approximated as the set of all grid points within the MSA on the resulting surface densities (one each for blacks, whites, and total population). To describe the racial composition of the environment around each point (egocentric neighborhoods), biweight kernel densities were calculated using varying bandwidths. Thus a 500-meter bandwidth describes the density of blacks (or whites) within a 500-meter radius circle of each point, with Gaussian-like distance decay. Any bandwidth can be specified allowing exploration of the role of the spatial scale of local environments on the calculation of metropolitan segregation indices. We initially considered 500-, 1000-, 2000-, and 4000-meter bandwidths which may approximate neighborhoods ranging from the small, walkable area around one's home (500 m) to a much broader sub-region of the MSA (4000 m) in which economic, educational and social transactions may occur [30,38,39]. We calculated spatial versions of the dissimilarity and the black isolation index using the formulas adapted from those proposed by Reardon & O'Sullivan [33]:(3)

Spatial dissimilarity index(4)

Spatial black isolation index

For each calculation, we summed values across all p points of the 50- by 50-meter grid within region R (the MSA). The total (black plus white) population density at each point, p, is indicated by *τ*_*p*_, while the proportion black is denoted either for the entire MSA (*π*), for a given point, p(*π*_*p*_), or for the spatial area surrounding point p as estimated from the kernel density function . One possible advantage of this flexible approach to the scale of the local areas in segregation measures is that other axes or dimensions of segregation may be seen as special cases of spatial evenness or isolation. Specifically Reardon argues that the clustering dimension of segregation is simply evenness (or unevenness) at a broader scale; therefore the dissimilarity index could approximate the traditional evenness dimension using 500-meter or 1000-meter bandwidths and may measure neighborhood clustering using larger bandwidths such as 4000-meters or greater [33].

### Measuring neighborhood segregation

Neighborhood-specific racial diversity (as opposed to MSA segregation) have most commonly been measured using the proportion black in a census tract. However the spatial density approach can be adapted to studies focused on neighborhood effects or analyses of point processes of geocoded health events. For instance the value of the kernel density for the grid point closest to a geocoded health event is an estimation of the racial composition (e.g. proportion black) in the spatial area (e.g. 500 m- to 4000 m- radius circle) surrounding that point with greater weight to points closer than those further away.

## Data sources

### MSA Segregation

We selected for analysis the 231 MSAs with a total population greater than 100,000 persons and greater than 5,000 black residents in the 2000 decennial census. Metropolitan statistical areas are defined as a central city of at least 50,000, and the surrounding counties which are deemed to be economically and socially integrated with the central city as determined in part by the proportion of outlying county residents who commute to the urban core for employment [40].

To determine the potential for mis-measurement of segregation in the context of a common epidemiologic analysis, we linked birth records to measures of segregation. All singleton live births born to non-Hispanic black or non-Hispanic white women residing in the eligible MSAs in 2000-2002 were abstracted from National Center for Health Statistics natality files [41]. These births are only coded at the MSA level, not for specific neighborhoods or tracts within each MSA. Births were classified as very preterm (gestational age at birth less than 32 weeks) or term (gestational age at birth 37 or more weeks). Very preterm birth was selected as a model outcome because it is a leading cause of infant mortality in the US [42], there is a large racial disparity in risk [43], and there is substantial geographic variation in the magnitude of the black-white disparity [44]. It therefore represents a significant public health problem that may be partially understood in terms of geographically varying exposures.

### Neighborhood segregation

To examine neighborhood-specific differences in measurement we used birth records from a single MSA, Atlanta, Georgia. The twenty-county Atlanta MSA has a large black population, and is notable for the size of the black middle class, and the growing importance of predominantly black and racially mixed suburbs outside the urban core.

We obtained from the Georgia Division of Public Health birth records for all singleton live births born to non-Hispanic black and non-Hispanic white women residing in the region in 2000-2002 with street-level geocodes. We measured the racial diversity environment of each mother in three ways: using the proportion black in the census tract of residence, using the previously calculated kernel density estimates for the grid point nearest the geocoded residence, and finally using the population weighted average of the kernel density points within each census tract, thus giving a spatial black isolation index for each census tract. This final step of re-aggregating the continuous surface information to the level of the areal census tract may better approximate the average racial diversity environment of each household within a tract by accounting for sub-tract micro-segregation, and incorporating information from adjoining tracts for residents near the border. It also allows exploration of differences between tract-derived and surface-density-derived segregation measures when the only available data are health events aggregated to the census tract level.

### Analysis

We calculated the mean of each index overall and by census region (Northeast, South, Midwest, West - see Table 1 for states within each region) for measures at all scales, and tested differences in the means across regions using ANOVA. We also calculated the mean and median of the arithmetic differences in segregation measures as the density-derived measure minus the tract-derived measure (e.g. MSA dissimilarity calculated with 500 m-egocentric neighborhoods minus MSA dissimilarity calculated with tracts). Because the surface-density-derived measures use a constant size neighborhood definition, differences in segregation indices at the metropolitan level could result from differences in the average size of census tracts within MSA's. Exploratory analysis suggested substantial variation in the median area of census tracts in MSA's and that this variation was strongly associated with census region and with the total MSA population count. Therefore we constructed plots of the arithmetic difference in segregation measures against the log of the MSA total population stratified on region.

**Table 1:** US Census Regions and States

Northeast	
Connecticut	New York
Maine	Pennsylvania
Massachusetts	Rhode Island
New Hampshire	Vermont
New Jersey	
Midwest	

Indiana	Missouri
Illinois	Nebraska
Iowa	North Dakota
Kansas	Ohio
Michigan	South Dakota
Minnesota	Wisconsin
South	

Alabama	Maryland
Arkansas	Mississippi
Delaware	North Carolina
District of Columbia	Oklahoma
Florida	South Carolina
Georgia	Tennessee
Kentucky	Texas Virginia
Louisiana	West Virginia
West	

Arizona	Nevada
California	New Mexico
Colorado	Oregon
Hawaii	Utah
Idaho	Washington
Montana	Wyoming

We fit a series of models to consider the impact that choice of segregation measure has on epidemiologic parameters of interest. Using national birth records we fit Poisson models to estimate the risk of very preterm birth across MSAs as a function of maternal race, metropolitan segregation and--recognizing previously reported racial differences in the effect of segregation--an interaction term between race and segregation. We changed the segregation measure entered with each model to include measures of dissimilarity and black isolation, estimated using tract-derived or spatial surface-density-derived (500-meter or 4000-meter egocentric neighborhoods) indices.

Segregation has variously been operationalized in the health literature as a continuous [45], or dichotomous/categorical variable [46]. Therefore we entered segregation into models as each. Following prior work, the threshold for dichotomizing MSAs as highly segregated versus not was 0.6 for dissimilarity and 0.7 for isolation [47]. In models with segregation as a continuous variable it was mean-centered to facilitate interpretation of model parameters. In other words after centering, an MSA with average segregation would have a value of zero, with less segregated MSAs below zero and more segregated MSAs above zero. We controlled for census region and MSA population size in all models. Risk ratios describe the relative excess risk for a full 1-unit change in segregation (e.g. index 1 vs 0).

We took a similar approach to quantifying the variation in model parameters for births to women in Atlanta neighborhoods. We fit logistic regression models with very preterm birth as the dependent variable, and maternal race, neighborhood segregation and an interaction term as independent variables. Because the evenness dimension is not meaningful for a single neighborhood, we only included measures of racial composition and density, which make up the isolation dimension.

All data analysis was conducted using R 2.9 [48].

## Results

### MSA segregation

**Figure 1:**
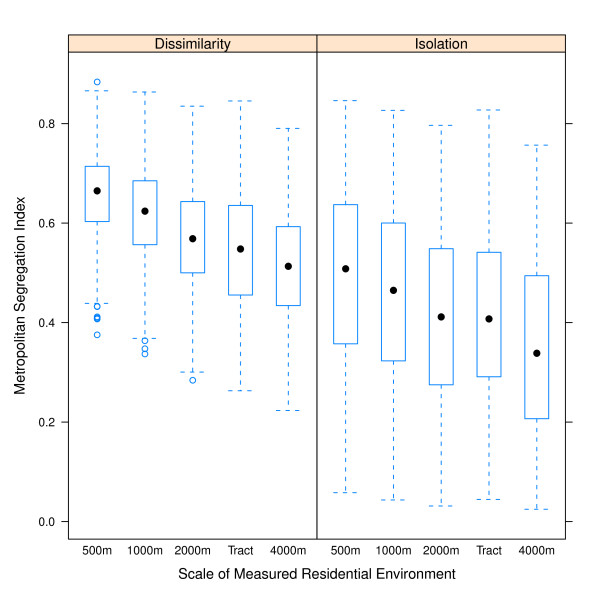
**Distribution of MSA residential segregation measured with five definitions of neighborhood environment**. Median value indicated by filled black dot, 25^th ^and 75^th ^percentile indicated by the lower and upper ends of the box, whiskers are 1.5 times the inter-quartile range, and outliers are hollow dots beyond whiskers.

**Figure 2:**
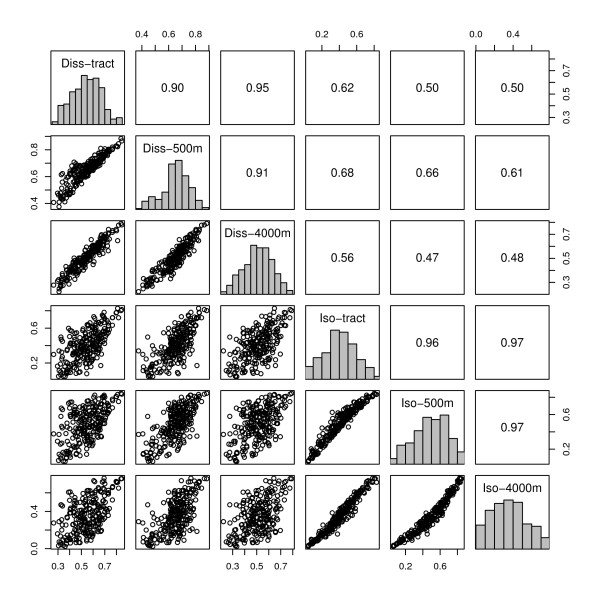
**Correlation of MSA black-white segregation indices**. Histograms (on the diagonal) and correlations (plots below diagonal, Spearman rank correlation coefficients above the diagonal) of segregation indices for 231 US MSA's, 2000. P-values for all pair wise correlations are < 0.0001. (Diss-tract: census tract-derived dissimilarity index; Diss-500 m, Diss-4000 m: surface-density-derived dissimilarity index for MSA's with neighborhoods defined with a 500 m or 4000 m kernel density function; Iso-tract: census tract-derived isolation index; Iso-500 m, Iso-4000 m: surface-density-derived isolation index for MSA's with neighborhoods defined with a 500 m or 4000 m kernel density function)

For the metropolitan dissimilarity index (evenness dimension of segregation) and the metropolitan isolation index (exposure dimension of segregation), the degree of segregation is highest when the operationalization of the local neighborhood environment is small (e.g. 500 m egocentric neighborhood) and gets progressively smaller with larger definitions of neighborhood (Table 2, Figure 1). As seen in Figure 1, on average census tracts appear equivalent to a 2000 m-egocentric neighborhood. Indices which differ by their definition of neighborhood scale are highly correlated (Figure 2). For the dissimilarity index the correlation is 0.9 to 0.95, and for isolation it is higher.

**Table 2:** Black-White residential segregation measures for 231 US Metropolitan Statistical Areas, 2000

			Census Region				
				
		Total	West	Midwest	Northeast	South	p-value^1^
Metropolitan Statistical Areas							
	N	231	33	52	35	111	
MSA Population	Mean	913,700	1,460,000	841,900	1,322,000	656,000	
	Median	436,100	753,200	405,000	629,400	319,400	
MSA % Black	Mean	13.3	4.5	9.7	8.3	19.2	
	Median	9.7	3.5	8.4	6.7	17.6	
Mean Segregation Index							
	Dissimilarity (500 m)^2^	0.652	0.531	0.688	0.708	0.652	< 0.001
	Dissimilarity (1000 m)^2^	0.612	0.490	0.655	0.676	0.608	< 0.001
	Dissimilarity (2000 m)^2^	0.566	0.453	0.615	0.640	0.553	< 0.001
	Dissimilarity (4000 m)^2^	0.512	0.414	0.568	0.588	0.490	< 0.001
	Dissimilarity (Tract)^3^	0.546	0.446	0.612	0.631	0.518	< 0.001
	Isolation (500 m)^2^	0.493	0.260	0.479	0.468	0.576	< 0.001
	Isolation (1000 m)^2^	0.457	0.235	0.445	0.433	0.536	< 0.001
	Isolation (2000 m)^2^	0.411	0.213	0.397	0.386	0.484	< 0.001
	Isolation (4000 m)^2^	0.356	0.186	0.338	0.322	0.426	< 0.001
	Isolation (Tract)^3^	0.409	0.228	0.415	0.413	0.458	< 0.001
Difference in Dissimilarity Index							
500 m - Tract	Mean	0.106	0.086	0.076	0.077	0.135	< 0.001
	25th percentile	0.068	0.061	0.057	0.052	0.095	
	Median	0.094	0.077	0.069	0.073	0.124	
	75th percentile	0.13	0.104	0.093	0.094	0.172	
4000 m - Tract	Mean	-0.034	-0.031	-0.044	-0.042	-0.028	0.04
	25th percentile	-0.055	-0.046	-0.064	-0.073	-0.014	
	Median	-0.033	-0.032	-0.045	-0.032	-0.027	
	75th percentile	-0.012	-0.016	-0.025	-0.01	-0.003	
Difference in Isolation Index							
500 m - Tract	Mean	0.084	0.031	0.065	0.056	0.119	< 0.001
	25th percentile	0.048	0.015	0.053	0.026	0.081	
	Median	0.073	0.03	0.068	0.053	0.111	
	75th percentile	0.111	0.04	0.074	0.076	0.154	
4000 m - Tract	Mean	-0.052	-0.042	-0.077	-0.091	-0.031	< 0.001
	25th percentile	-0.073	-0.057	-0.096	-0.127	-0.049	
	Median	-0.046	-0.036	-0.072	-0.093	-0.027	
	75th percentile	-0.024	-0.031	-0.054	-0.056	-0.01	

1. P-value from ANOVA analysis of mean values across census regions.

2. Dissimilarity and isolation indices calculated with a spatial egocentric neighborhood defined by biweight kernel with specified bandwidth.

3. Dissimilarity and isolation indices calculated with the census tract as the local/neighborhood area of interest

The degree of residential segregation varies significantly by region of the country (Table 2). While the Western MSA's have the highest median population count, they also have the lowest proportion black, and the lowest average values on all measures of black-white segregation. In contrast the Southern MSA's have the smallest median population count, the highest proportion black, but not necessarily the highest segregation. Isolation (which is sensitive to the MSA proportion black) is in fact highest on average in the South, but dissimilarity is lower in the South than either Northeastern or Midwestern MSA's across all neighborhood definitions.

The average arithmetic differences between segregation indices also vary by region. The largest differences comparing small egocentric neighborhoods (500 m) to census tracts is in the Southern MSA's for both the isolation (mean change 0.12) and dissimilarity (mean change 0.14) indices. In contrast the largest difference for dissimilarity between large egocentric neighborhoods (4000 m) and census tracts is in the Midwestern MSA (mean change -0.04), but for isolation the largest difference is in the Northeastern MSAs (mean change -0.09).

**Figure 3:**
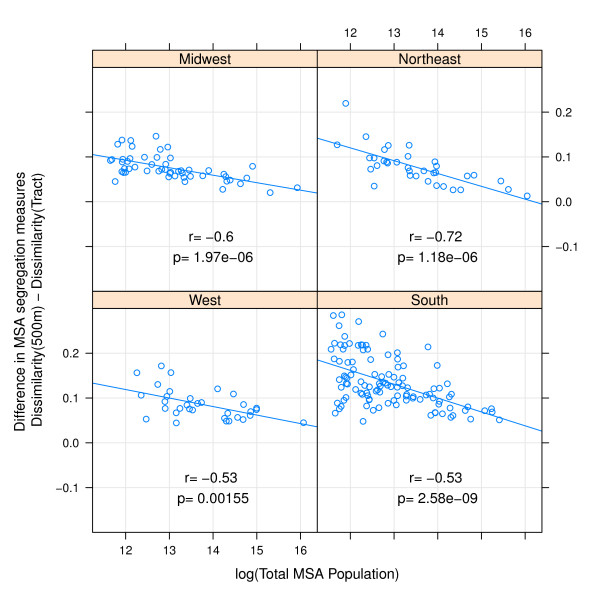
**Difference in MSA dissimilarity index when measured using 500 m-egocentric neighborhood versus census tract by region**. The arithmetic difference of MSA segregation calculated with 500-meter bandwidth surface-density-derived dissimilarity index as compared with census-tract derived dissimilarity is plotted on the y-axis; log of MSA total population count in 2000 is plotted on x-axis. Pearson correlation coefficient is 'r', and corresponding p-value. Line is best fit linear regression. Panels represent MSA's within each of four census regions.

**Figure 4:**
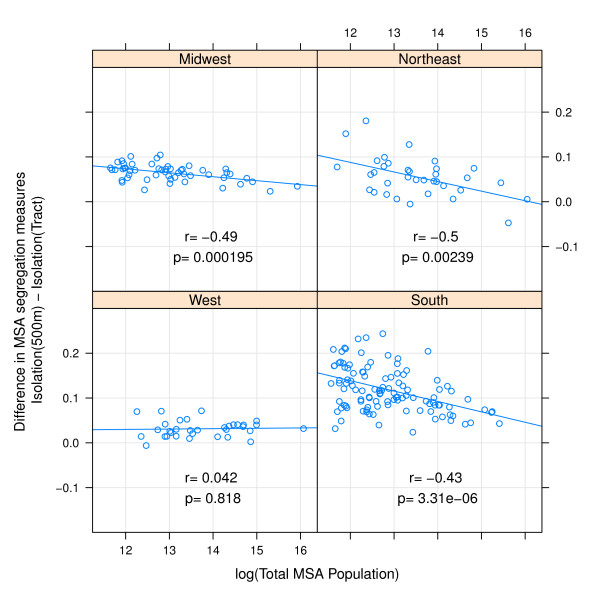
**Difference in MSA isolation index when measured using 500 m-egocentric neighborhood versus census tract by region**. The arithmetic difference of MSA segregation calculated with 500-meter bandwidth surface-density-derived isolation index as compared with census-tract derived isolation is plotted on the y-axis; log of MSA total population count in 2000 is plotted on x-axis. Pearson correlation coefficient is 'r', and corresponding p-value. Line is best fit linear regression. Panels represent MSA's within each of four census regions.

**Figure 5:**
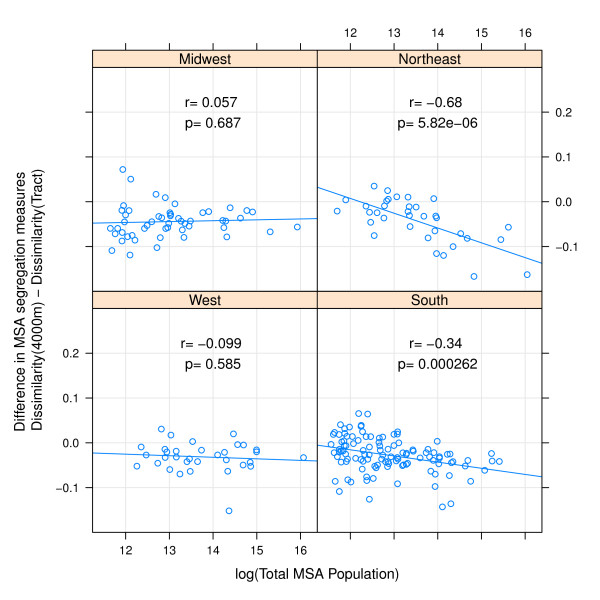
**Difference in MSA dissimilarity index when measured using 4000 m-egocentric neighborhood versus census tract by region**. The arithmetic difference of MSA segregation calculated with 4000-meter bandwidth surface-density-derived dissimilarity index as compared with census-tract derived dissimilarity is plotted on the y-axis; log of MSA total population count in 2000 is plotted on x-axis. Pearson correlation coefficient is 'r', and corresponding p-value. Line is best fit linear regression. Panels represent MSA's within each of four census regions.

**Figure 6:**
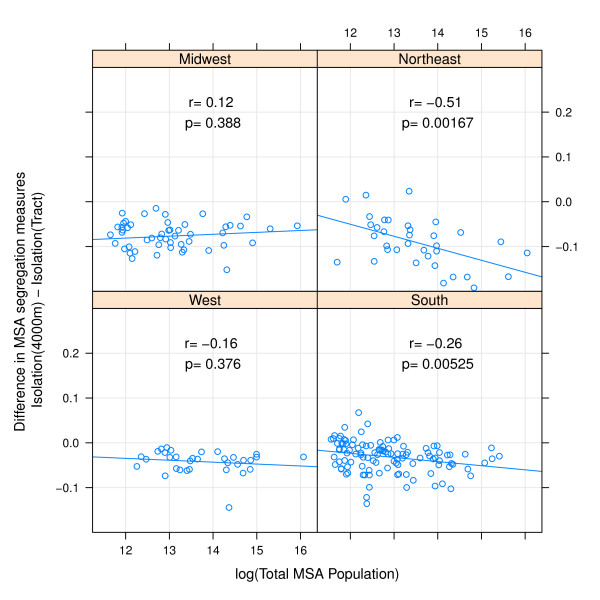
**Difference in MSA isolation index when measured using 4000 m-egocentric neighborhood versus census tract by region**. The arithmetic difference of MSA segregation calculated with 4000-meter bandwidth surface-density-derived isolation index as compared with census-tract derived isolation is plotted on the y-axis; log of MSA total population count in 2000 is plotted on x-axis. Pearson correlation coefficient is 'r', and corresponding p-value. Line is best fit linear regression. Panels represent MSA's within each of four census regions.

In addition to systematic differences by regions, there are also important differences in measurement change by MSA population size. Comparing small egocentric neighborhoods to census tracts, the difference in either isolation or dissimilarity decreases with increasing population size in all regions with the exception of isolation in Western MSAs (Figures 3 &4). The differences are less consistent when comparing 4000 m egocentric neighborhoods to tracts where the strongest association with population size is evident in the Northeastern MSA's (Figures 5 &6).

Table 3 reports results from Poisson models of the association between segregation and very preterm birth across 231 MSAs. Parameters of interest include the independent association of segregation with very preterm birth using each segregation index, the magnitude of the black-white disparity with inclusion of segregation, and the interaction between maternal race and segregation. Because race is coded black = 1 and white = 0, the main effect of the segregation parameter represents the relative change in risk for white mothers exposed to a highly segregated MSA as compared to a minimally segregated MSA. Within dimensions of segregation the estimated risk ratio is negligible and varies modestly, ranging from 0.91 to 0.95 for continuously measured dissimilarity, from 0.91 to 0.96 for isolation, and similarly for dichotomized segregation indices.

**Table 3:** Association of MSA segregation and very preterm birth among Black and White mothers, 231

**MSAs**										
	**Main effect of segregation**			**Main effect of race**			**Segregation × Race Interaction**			**Model Fit (Deviance)**
				
	**RR**	**95% CI**		**RR**	**95% CI**		**RR**	**95% CI**		
	
**BASELINE**				3.11	3.07	3.15				2104
**Continuous**										
Dissimilarity										
Tract	0.91	0.83	1.00	2.96	2.92	3.01	1.68	1.52	1.87	1990
500 m	0.95	0.85	1.07	2.96	2.92	3.01	2.12	1.84	2.43	1948
4000 m	0.95	0.87	1.05	2.99	2.94	3.03	1.69	1.50	1.89	2001
Isolation										
Tract	0.91	0.86	0.96	2.91	2.86	2.96	1.51	1.40	1.62	1967
500 m	0.96	0.91	1.01	2.91	2.87	2.96	1.52	1.41	1.64	1963
4000 m	0.93	0.88	0.98	2.93	2.88	2.98	1.44	1.34	1.54	1990
										
**Binary^†^**										
Dissimilarity										
Tract	0.97	0.95	0.99	2.91	2.85	2.97	1.11	1.08	1.14	2038
500 m	0.98	0.95	1.01	2.80	2.69	2.91	1.12	1.08	1.17	2067
4000 m	0.97	0.95	0.99	2.99	2.94	3.05	1.08	1.06	1.11	2062
Isolation										
Tract	0.96	0.93	0.98	3.00	2.95	3.04	1.17	1.13	1.20	1978
500 m	0.96	0.94	0.98	2.97	2.92	3.01	1.13	1.10	1.16	2011
4000 m	0.91	0.88	0.94	3.03	2.99	3.07	1.18	1.14	1.22	2022

NOTE: Race is coded Black = 1, White = 0, and all continuous segregation indices are mean-centered so that the main effect of segregation is the effect for white women only, and the main effect of race is the excess relative risk for black as compared with white women at average segregation levels (e.g. mean-centered segregation equals zero for in an MSA with average level of segregation). The interaction term is then the excess relative risk for black women in the most compared with the least segregated MSAs. All models are adjusted for census region and MSA population size.

† Dissimilarity index dichotomized into low (< 0.6) = 0, and high (≥0.6) = 1. Isolation index dichotomized at 0.7

Data source: National Center for Health Statistics; all singleton live births to non-Hispanic white and non-Hispanic black mothers in 231 US MSA's, 2000-2002

In contrast to the very small differences in effect-size estimates for white women, the interaction term between race and segregation (indicating the excess relative risk experienced by black women above and beyond the main effect of exposure to high versus low segregation) varies more widely. The risk ratio for the segregation-race interaction term is 1.68, 2.12, and 1.69 when segregation is measured using tract-derived dissimilarity, 500-meter or 4000-meter surface-density-derived dissimilarity respectively. This represents a 39% change in the size of the effect estimate. When segregation is modeled as a binary hi/low variable the effect across all measures is smaller but still variable by choice of index. The results of the interaction term are most interpretable when combined with the independent effects of race and segregation. As discussed the independent effect of segregation varied little by measure, but the independent effect of race (the excess relative risk for very preterm birth among black as compared with white mothers) was most variable when dissimilarity segregation was modeled as a dichotomous variable, where risk ratios range from 2.80 to 2.99, a measured effect estimate change of 9.5%.

The deviance from each model is a measure of relative fit with smaller values suggesting better fit. Comparing each segregation measure to the baseline model which included only race, region, and population size, the largest reduction in deviance (best fit) occurs with dissimilarity or isolation measured with 500-meter egocentric neighborhoods (change in deviance is 156 for dissimilarity, and 141 with isolation).

### Neighborhood segregation

**Figure 7:**
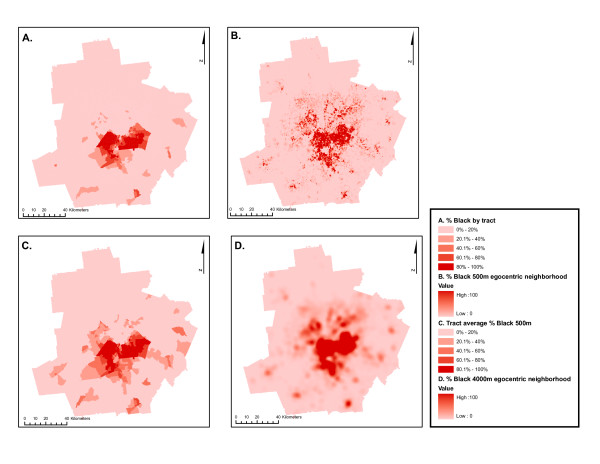
**Residential area racial composition of sub-areas in Atlanta MSA, 2000, using alternate measures**. Twenty-county Atlanta MSA distribution of black residents as measured with four methods: A: % black in census tract; B. surface-density-derived proportion black using 500-meter bandwidth kernel; C. population-density-weighted average of 500-meter bandwidth proportion black aggregated to the census tract; D. surface-density-derived proportion black using 4000-meter bandwidth kernel.

Figure 7 displays maps of the neighborhood racial composition using each method of measuring segregation in neighborhoods across the Atlanta MSA. Panel A is the proportion black in each census tract; panel B is the results of surface-density-derived local proportion black using 500-meter egocentric neighborhoods; Panel C is the resulting segregation values when the 500-meter surface-density-derived values are aggregated to the level of census tracts using population-density weighting; panel D is the 4000-meter surface-density-derived pattern. Most black residents of Atlanta live in the central and central-southern portion of the MSA, although the pattern varies somewhat by measure. The micro-segregation within tracts is most visible using the 500-meter measure, and it is likely this micro-segregation within tracts which results in the increased number of mixed-race and predominantly black tracts in panel C compared to A.

Table 4 summarizes results from logistic regression models of the association between neighborhood racial composition and odds for very preterm birth among black and white mothers. In contrast to MSA segregation, the main effect of segregation suggests the odds for very preterm birth increase for white mothers as neighborhood proportion black increases. For black mothers, the odds of very preterm birth also increases as neighborhood proportion black increases, but it does so less steeply than for whites, as demonstrated by the odds ratio for the interaction effect below the null value of 1.0. In other words, the 'protective' interaction term describes the tempering of the main effect of segregation experienced by black women, but in the case of each segregation index, the main effect is so strongly deleterious that the net result is still higher risk for blacks and for whites as segregation increases.

**Table 4:** Association of neighborhood racial composition and very preterm birth among Black and White

**mothers, Atlanta, GA**										
	**Main effect of segregation**			**Main effect of race**			**Segregation × Race Interaction**			**Model Fit (Deviance)**
				
	**OR**	**95% CI**		**OR**	**95% CI**		**OR**	**95% CI**		
	
**BASELINE**				2.84	2.70	2.99				62845
**Continuous**										
Tract % black	1.75	1.35	2.24	2.45	2.28	2.64	0.69	0.53	0.90	62813
500-m % black	1.59	1.30	1.95	2.43	2.26	2.62	0.75	0.60	0.94	62813
4000-m % black	1.68	1.34	2.09	2.47	2.30	2.65	0.71	0.56	0.91	62814
Tract-500-m^†^	1.68	1.34	2.08	2.45	2.28	2.63	0.71	0.56	0.91	62811
										
**Trichotomous**										
Tract % black										
< 0.3	1.00	--	--	2.75	2.55	2.96	1.00	--	--	62818
0.3-0.7	1.27	1.11	1.44				0.80	0.69	0.94	
> 0.7	1.22	0.85	1.67				0.93	0.67	1.34	
500-m % black										
< 0.3	1.00	--	--	2.75	2.53	2.99	1.00	--	--	62811
0.3-0.7	1.25	1.10	1.42				0.81	0.69	0.95	
> 0.7	1.50	1.20	1.85				0.74	0.59	0.94	
4000-m % black										
< 0.3	1.00	--	--	2.69	2.48	2.92	1.00	--	--	62814
0.3-0.7	1.23	1.10	1.38				0.87	0.76	1.01	
> 0.7	1.43	1.10	1.82				0.81	0.62	1.06	
Tract-500-m^†^										
< 0.3	1.00	--	--	2.80	2.59	3.03	1.00	--	--	62811
0.3-0.7	1.31	1.16	1.47				0.76	0.66	0.88	
> 0.7	1.27	0.96	1.64				0.88	0.67	1.17	

NOTE: Race is coded Black = 1, White = 0, and all continuous segregation indices are mean-centered so that the main effect of segregation is the effect for white women only, and the main effect of race is the excess relative risk for black as compared with white women at average segregation levels (e.g. mean-centered segregation equals zero for an neighborhood with average racial composition). The interaction term is then the excess relative risk for black women in the most compared with the least segregated neighborhoods.

†Tract-500-m are obtained by taking a population-weighted average of the racial composition in the 500-meter vicinity of each grid point within a census tract, thus approximating the average spatial isolation experienced by residents of each tract.

Data source: Georgia Department of Community Health, Office of Health Indicators for Planning; all singleton live births to non-Hispanic black and non-Hispanic white mothers in Atlanta MSA, 2000-2002

For both continuous and categorical parameterizations of segregation, there is important variation in effect size estimates depending on the measure chosen. For instance the odds ratio for high versus low segregation measured continuously at the tract level is 1.75 (95% CI 1.35-2.24), while the odds ratio for 500-meter surface-density-derived isolation is 1.59 (1.30-1.95), a 21% change in effect-size estimate; the magnitude of the interaction term is similarly attenuated. When neighborhoods are categorized into three groups, the effect-size estimate for high segregation (> 0.7) as compared with low segregation (< 0.3) varies from an OR of 1.50 when segregation is measured with 500-meter surface-density-derived isolation to 1.22 when measured with tract-level percent black.

## Discussion

As public health researchers increasingly look to structural or upstream explanations for widespread racial health disparities there is increasing need for better tools to measure and describe residential segregation. Epidemiologists, demographers, and medical geographers have tested hypotheses about the role of residential segregation on a wide variety of health outcomes, but with very little attention paid to possible measurement error or misclassification with regards to exposure to segregation. The question of misclassification in epidemiologic research is crucial because of the likelihood of imprecise or biased estimates of the parameters of interest in the presence of measurement error [49,50].

We find that the class of surface-density-derived measures of evenness and isolation segregation proposed by Reardon, et al, is highly correlated with traditional census tract-derived indices. Although reassuring, the high overall degree of correlation between measures may obscure important differences of interest to health researchers considering segregation as an exposure or covariate. Understanding the potential impact of measurement differences in the context of social epidemiology or health geography requires recognition of the causes for patterns of measurement difference, the relevance of neighborhood scale in conceptualizing residential segregation, and the degree and direction of measurement difference for health outcomes.

Census tracts were created for specific administrative purposes with the goals of homogeneity of population and a semblance of consistency in population size. It is not surprising that the area and population density of tracts varies not only within MSA's but also across regions of the US and with respect to overall population size. With increasing total population size, population density may increase and thus tracts are more homogenous in size; this may explain the parity of surface-density-derived segregation measures with tract-derived measures in the most populated urban areas. In contrast for studies focusing on smaller MSAs or particularly on MSAs in the Southern US, there appears to be increased likelihood of differences in measured segregation. One implication of these patterns is that future research should consider variation in segregation-health associations within and between regions and MSAs categorized by population size.

The finding that these differences are greater for dissimilarity than for isolation point to differences in the two constructs. Dissimilarity measures relative evenness or uniformity of the distribution of blacks across neighborhoods and is therefore insensitive to absolute size of the black population. High isolation on the other hand is only possible in cities with a sufficiently large black population to allow complete spatial separation of blacks from whites. Therefore isolation is somewhat correlated with population size, and the largest cities had the smallest differences in measures. There are also differences between dissimilarity and isolation across regions of the US. The higher isolation for Southern MSAs is consistent with the relatively larger black population in Southern cities, but the fact that Northern and Midwestern MSAs have higher dissimilarity than the South may result from those MSAs being larger on average and thus have greater spatial separation between racial groups (more unevenness). Although isolation and dissimilarity are correlated these two constructs can be useful together in understanding what aspect of the segregation pattern is most health relevant [21,24].

The question of the importance of spatial scale for conceptualizing neighborhoods in health research is not new [51,52]. Rather than recommending a single 'ideal' scale many investigators argue for exploring place-health associations at a variety of scales to understand how associations are spatially patterned [53,54]. In that regard census tracts are not aspatial, they are simply of a somewhat arbitrary scale which appears to correlate in these MSA's with a 2000m-egocentrically defined neighborhood (Figure 1). Thus segregation indices estimated using only census tracts may miss opportunities to more richly describe associations between health and residential patterns at different scales.

In this study the largest differences between tract-derived measures and surface-density derived measures was for the smallest egocentric neighborhood considered, defined as a 500-meter-radius circle around each residence. Although this spatial scale (and in fact all of the egocentric scales assessed) are arbitrary and imperfect descriptions of experienced residential environment, a 500-meter area may be particularly meaningful for some aspects of residential segregation. Sastry, et al found that the majority of residents surveyed in sprawling Los Angeles County self-reported their neighborhood to be either the street or block on which they lived, or the area within a 15-minute walk of their home [39]. This is similar to the area approximated by a 500-meter radius egocentric neighborhood. A study of residents' perception of neighborhood safety and security in Flint, Michigan evaluated the association between proximity to deteriorating residential housing quality and fear of crime, quality of social capital and general satisfaction [55]. The investigators found that the resident's fear of crime and perception of low social capital was greatest when they lived 0.25 miles (about 400 meters) away from deteriorating residential housing.

Ultimately researchers should be interested in whether their choice of segregation measure results in biased or unnecessarily imprecise estimates of the association of place and health. As an example, we find that when comparing different measures of segregation, the estimated effect of MSA segregation on very preterm birth varies in magnitude (but not direction) particularly for black mothers, where the smallest neighborhood approximation had the strongest association. In contrast for studies focusing on neighborhood racial composition, we find that the choice of measure changes the magnitude of the segregation effect for white women, and to lesser degree black women. Using continuously measured indices, the strongest association was found using proportion black within census tracts, although when segregation was categorized the largest effect of high versus low segregation was for the 500-meter egocentric neighborhoods.

While these models are not adjusted for other important confounders--because the object of the current analysis is variation in the segregation parameter, not causal inference with regards to the parameter--several patterns are worthy of mention. The difference in association for white women in this neighborhood analysis as compared with the MSA-level analysis speaks to one of the critical distinctions between segregation studies where the spatial unit of observation is the neighborhood as opposed to the MSA. The characteristics of the residential environment which are toxic to health (e.g. crumbling infrastructure, concentrated poverty, high crime) are bad for all residents, black or white, as demonstrated in the Atlanta neighborhood study where white and black women have elevated very preterm birth risk as segregation increases. What may account for the differences in the MSA analysis, however, is that as segregation increases, the probability of white women experiencing those unhealthy neighborhoods declines, so that in highly segregated MSAs even poor white women tend to live in mixed income neighborhoods, while poor blacks predominantly live in high-poverty neighborhoods [22].

## Limitations

There are several important limitations to our study. First, we used census blocks as the demographic input from which we created surface-densities of populations. Census blocks are the highest resolution data available, but the possibility for imprecise estimation of fine-scale residential density remains.

Secondly, we focused primarily on black-white residential segregation as this particular pattern has most consistently been associated with health disparities in the US [21]. While the segregation indices are valid descriptions of black-white residential patterns, other population patterns may be of interest to researchers as well, including multi-group segregation among Hispanics, whites, and blacks, or economic segregation overall or within racial/ethnic groups. The spatial surface density approach to measuring segregation can be extended to each of these cases [56,57].

Third, in the example of residential segregation and very preterm birth we chose to use simple models, with the focus on the relative change in the segregation parameters as the index of segregation was changed. A consequence is that several important confounders of the association were ignored. While our findings should not be interpreted as describing causal associations, in other work we confirm that these patterns persist in more complex multi-level models with adjustment for individual and area-level covariates (manuscript under review).

Finally, because national birth records are not available with small-area geocodes, we were not able to simultaneously consider neighborhood and MSA-level segregation, and thus cannot make inference as to which scale is most responsible for the observed associations.

## Conclusions

Social epidemiologists, health demographers, and medical geographers are increasingly interested in the association between patterns of racial residential segregation and health disparities. As this area evolves more nuanced approaches to measuring segregation are needed. We find that a new class of explicitly spatial segregation measures is highly correlated with census tract derived measures, but specific measures differ in systematic ways which may be relevant to particular studies. Specifically there are systematic variations in the values of the dissimilarity and isolation indices among US Census regions and between MSAs with different population sizes. Investigators should consider these variations in future research. Further research is also needed to better understand the role of spatial scales in describing the relationship between segregation and health, noting that relevant scales may vary with different health outcomes. In fact exploring variations in the place-health associations across spatial scales may be considered an additional dimension of the residential context.

While we find meaningful differences in the magnitude of effect estimates between segregation and very preterm birth, further research is needed to determine whether choice of segregation measure is more or less important for other health outcomes.

## Competing interests

The authors declare that they have no competing interests.

## Authors' contributions

MRK conceived of the study, performed all GIS calculations, conducted statistical analyses, and lead the writing of the manuscript. HLC, CDD-B, CRH, and LAW each provided substantial input in guiding the analysis, interpretation of results, and each contributed to the preparation of the manuscript. All authors have read and approved the final manuscript.
